# Identification and functional analysis of long intergenic noncoding RNA genes in porcine pre-implantation embryonic development

**DOI:** 10.1038/srep38333

**Published:** 2016-12-06

**Authors:** Jingyu Li, Zhengling Gao, Xingyu Wang, Hongbo Liu, Yan Zhang, Zhonghua Liu

**Affiliations:** 1 College of Life Science, North-east Agricultural University, Harbin, 150030, China; 2 Chong Qing Reproductive and Genetics Institute, Chongqing Obstetrics and Gynecology Hospital, 64 Jing Tang ST, Yu Zhong District, Chongqing, 400013, China; 3 College of Bioinformatics Science and Technology, Harbin Medical University, Harbin, 150080, China

## Abstract

Genome-wide transcriptome studies have identified thousands of long intergenic noncoding RNAs (lincRNAs), some of which play important roles in pre-implantation embryonic development (PED). Pig is an ideal model for reproduction, however, porcine lincRNAs are still poorly characterized and it is unknown if they are associated with porcine PED. Here we reconstructed 195,531 transcripts in 122,007 loci, and identified 7,618 novel lincRNAs from 4,776 loci based on published RNA-seq data. These lincRNAs show low exon number, short length, low expression level, tissue-specific expression and *cis*-acting, which is consistent with previous reports in other species. By weighted co-expression network analysis, we identified 5 developmental stages specific co-expression modules. Gene ontology enrichment analysis of these specific co-expression modules suggested that many lincRNAs are associated with cell cycle regulation, transcription and metabolism to regulate the process of zygotic genome activation. Futhermore, we identified hub lincRNAs in each co-expression modules, and found two lincRNAs *TCONS_00166370* and *TCONS_00020255* may play a vital role in porcine PED. This study systematically analyze lincRNAs in pig and provides the first catalog of lincRNAs that might function as gene regulatory factors of porcine PED.

A vast amount of long intergenic noncoding RNAs (lincRNAs) in various species are being identified by increasingly large-scale RNA-sequencing (RNA-seq) projects^1^,^2^,^3^,^4^. Several researches have demonstrated that some lincRNAs play important roles in various biological processes, such as epigenetic regulation^5^,^6^, maintenance of pluripotency^7^,^8^, and transcriptional regulation^9^,^10^. Pig is an ideal model for reproduction and biomedical applications owing to their morphological and functional similarities with humans^11^,^12^, thus a comprehensive genome-wide identification of lincRNAs is required. To date, for genome-wide identification across various tissues, there are only one study indentified 6,621 lincRNAs through Coding Potential Calculator (CPC) tool^13^, which classify long noncoding transcripts based on putative ORF or peptide hits^14^. However, for reconstructed from high-throughput sequencing data of imcomplete annotated species like pig, using tools which distinguish protein-coding and noncoding transcripts independent of known annotations might be more suitable.

It is well known that genome-wide gene activation in the zygote, termed zygotic genome activation (ZGA), is crucial for successful pre-implantation embryonic development (PED)^15^. Therefore, understanding the molecular mechanism underlying ZGA is required. Although the transcription of lincRNAs have been extensively investigated in mouse and human PED^2^,^16^,^17^, little is known about its function. In pig, ZGA mainly occurs between the 4-cell and 8-cell stages^18^. Because few studies exist to describe the transcriptome changes in porcine PED, functional research about lincRNAs in porcine PED is limited.

In this study, we performed comprehensive genome-wide characterization of novel lincRNAs of various tissues and identified 7,618 novel lincRNAs from 4,776 loci. We also systematically analyzed genomic signatures, expression patterns and regulatory modules of all lincRNAs. To investigate the potential roles of lincRNAs in porcine PED, we performed weighted gene co-expression network analysis (WGCNA)^19^, and revealed that many lincRNAs show strong correlation with specific developmental stages. In addition, we identified the hub lincRNAs in the co-expression network, and found two hub lincRNAs showed specific expression in reproductive tissues and the ZGA process, which might play important roles in porcine PED. We believe our genome-wide annotation of lincRNAs would help on a better understanding of molecular regulations that occur in porcine PED.

## Results

### Identification of 7618 lincRNAs based on RNA–seq Data Sets in pig

**Figure 1:**
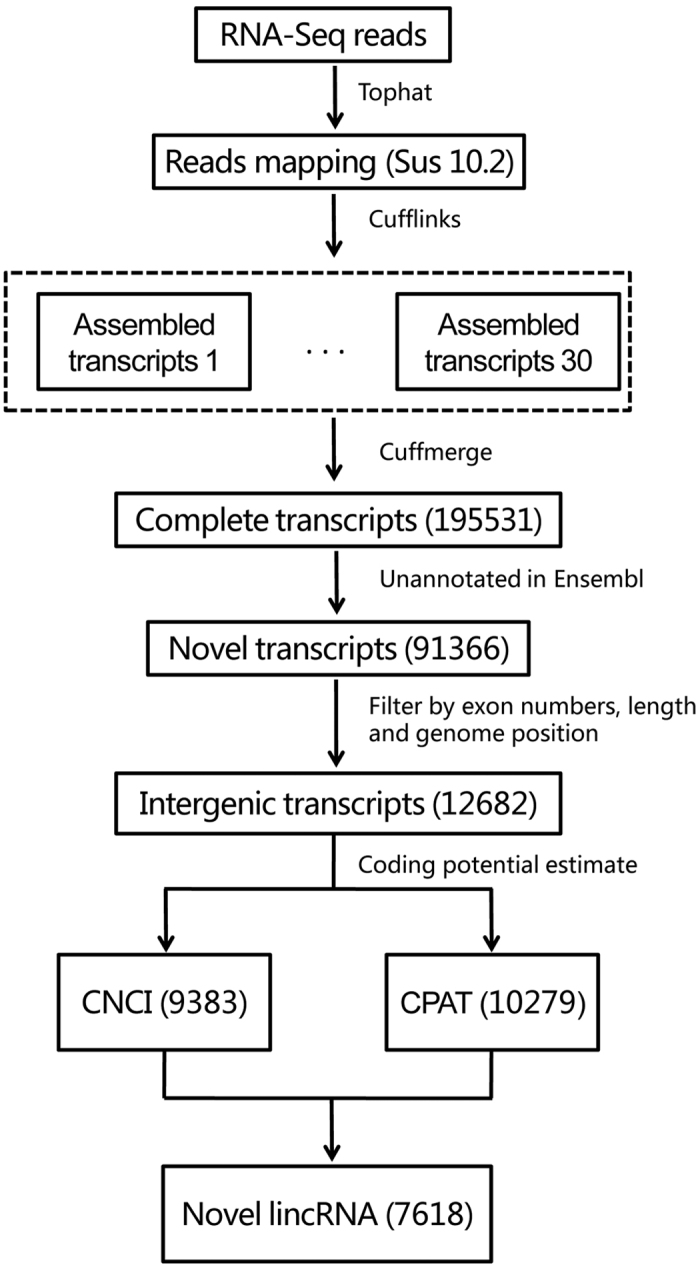
Overview of pig lincRNAs identification pipeline.

To comprehensively identify pig lincRNAs, we used five RNA-seq data sets involving various tissues of the pig (Supplementary Table S1)^13^,^20^,^21^,^22^,^23^. We developed a pipeline to identify novel lincRNAs as shown in Fig. 1. Briefly, all reads were aligned to the pig genome Sus scrofa 10.2 using Tophat^24^. Then, the mapped reads of each data were assembled into one set of transcripts with cufflinks^24^. The reconstructed transcripts for different data were merged into a sigle nonredundant transcript set using the Cuffmerge provided by Cufflinks. We identified 195,531 transcripts originating from 122,007 gene loci. Based on our identification pipeline, we removed known mRNAs recorded in Ensembl databases. resulting in a data set containing 91,366 novel transcripts. Then, we applied a strict criteria to define the intergenic transcripts as following: (1) The exon number must ≥2, 2) length should be ≥200 nt, and 3) genomic coordinates must be at least 500 bp away from any genes annotated in the Ensembl Sus scrofa10.2, a set of 12,682 intergenic transcripts was obtained. Finally, we used two different methods, CNCI^25^ and CPAT^26^, to evaluate the protein-coding potential, and obtained 7,618 lincRNAs encoded by 4,776 gene loci.

### Structure features of pig lincRNAs

**Figure 2:**
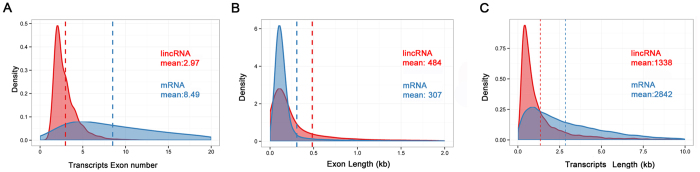
Features of pig lincRNAs. (**A**) Exon number distribution of transcripts for all lincRNAs and protein-coding transcripts. (**B**) Exon length distributions of lincRNA and protein-coding transcripts. (**C**) Transcript length distributions of lincRNA and protein-coding transcripts. Red: lincRNA. Blue: protein-coding transcripts.

Previous studies in human or mouse have shown that there were many difference between lincRNAs and protein-coding genes, such as exon number, exon length and transcript length^27^,^28^,^29^. Thus we compared our predicted lincRNAs with mRNAs recorded in Ensembl to determine whether pig lincRNAs are characterized by these features. Even though we filtered all unspliced lincRNAs, lincRNAs show a striking tendency to have fewer exons than protein-coding transcripts (mean 2.97 and 8.49 exon, respectively; Kolomogorv-Smirnov Test, *P*-value < 2.2 × 10^−16^) (Fig. 2A). While pig lincRNA exons were on average longer than those of protein-coding transcripts (mean 484 and 307 bp, respectively; Kolomogorv-Smirnov Test, *P*-value < 2.2 × 10^−16^) (Fig. 2B), which is consistent with previous reports from GENCODE^3^. Because of the fewer exons, overall lincRNA transcripts are shorter than protein-coding transcripts (mean 1338 bp and 2842 bp. respectively; Kolomogorv-Smirnov Test, *P*-value < 2.2 × 10^−16^) (Fig. 2C).

### Low expression and tissue-specificity of pig lincRNAs

**Figure 3:**
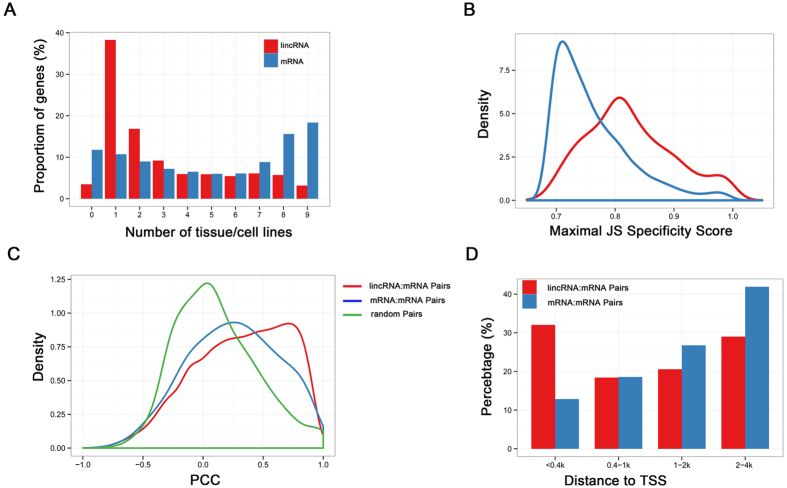
Characteristics of pig lincRNAs expression. **(A**) Distribution of the number of tissues in which lincRNA and protein-coding transcripts are detected (FPKM >0.1). (**B**) Distribution of maximal tissue specificity scores in various categories. lincRNA (Red); mRNA (Blue) (**C**) Distribution of correlation of neighbouring (gene body distance <10 kb). (**D**) Distribution of distance between 2 TSS of neighbour gene pairs. lincRNA: Coding gene pairs (Red); Coding gene pairs (Blue); Random gene pairs (Green).

We investigated the expression patterns of lincRNAs using RNA-seq data sets from various tissues and cell lines. As shown previously in other mammalian species^13^,^16^,^27^,^29^,^30^,^31^, we also found the expression levels of lincRNA were generally lower than protein-coding genes in pig (see Supplementary Fig. S1). Almost 38% of lincRNAs were only detected in a single tissue compared with 10% of protein-coding genes using an FPKM threshold greater than 0.1 (Fig. 3A), which suggested that porcine lincRNAs are more variable than protein-coding transcripts. To quantitatively assess the expression specificity of each transcript, we applied an entropy-based metric that relies on Jensen-Shannon distance-based algorithm to calculate expression specificity score of each transcript^27^. Consistent with our previous observation^3^,^16^,^17^, we have also found that our predicted pig lincRNAs show higher JS scores on average than protein-coding genes (mean 0.83 and 0.76; Kolomogorv-Smirnov Test, *P*-value < 2.2 × 10^−16^) (Fig. 3B). Together, these results suggested pig lincRNAs show lower and more tissue-specific expression than protein-coding genes.

### The potential *cis*-acting correlation between pig lincRNAs and their neighbouring protein-coding genes

Several studies have indicated that lincRNAs may act in either positive or negative way to regulate the expression of neighboring protein-coding genes^32^,^33^. To determine whether pig lincRNAs share the similar regulation patterns, we focused on the gene pairs whose minimal distance was within 10 kb^16^. Interestingly, using Gene Ontology (GO) analysis based on the DAVID web server, we found that these neighboring protein-coding genes of pig lincRNAs enriched in “regulation of transcription” function and nucleus compartment (Supplementary Table S2). Then, we analysed the correlation of the expression patterns between the lincRNAs and their neighboring protein-coding genes. Based on the expression levels across five RNA-seq data sets, we found a higher correlation in lincRNA:mRNA pairs than mRNA:mRNA pairs (mean 0.315 and 0.252, both significantly higher than random, 0.113; Kolomogorv-Smirnov Test, *P*-value < 2.2 × 10^−16^) (Fig. 3C). Previous studies have observed many transcriptions of lincRNAs within 4 kb around the transcription start sites (TSSs) of protein-coding genes and their coordinated expression pattern^34^,^35^. We analyzed the TSSs distance between lincRNAs and their neighbouring protein-coding genes, in total, we found 462 lincRNA:mRNA pairs whose TSSs distance were within 4 kb. It is worth noting that 32% of lincRNAs in lincRNA:mRNA pairs were originate within 400nt, which compared to 12% of TSSs distance in mRNA:mRNA pairs (Fig. 3D), might explaining the higher regulation ability in *cis* in lincRNAs.

Taken together, these analyses revealed that lincRNAs in pig could act in *cis* to regulate the expression of their neighboring protein-coding genes, and these lincRNAs which significantly regulate their neighbors might represent interesting candidates to be tested in further experimental studies.

### Function anlaysis of pre-implantation embryonic development associated lincRNAs

**Figure 4:**
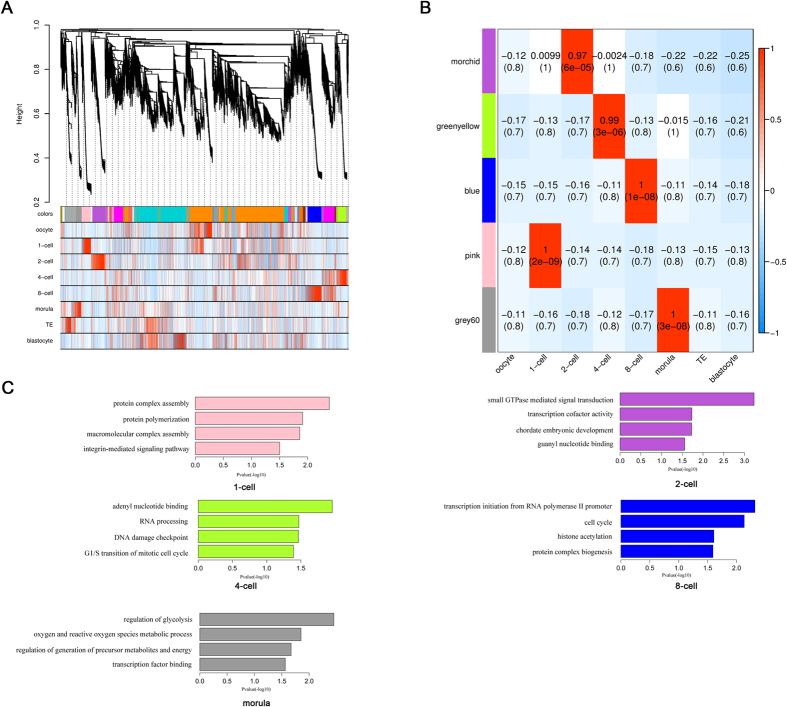
Function prediction of PED associated lincRNAs. **(A**) Hierarchical cluster tree of co-expression modules identified by WGCNA. Modules correspond to branches and are labelled by colours as indicated by the first colour band underneath the tree. Upper color panel: module membership of genes; Bottom color panel: correlation between transcripts and particular stage: High correlation (Red); Low correlation (Blue), Median correlation (White). (**B**) Stage specific co-expression gene modules and their correlation to development stage. Numbers of each cell represent correlation of module and development stage, and *p*-value of each correlation value. Color of each square is correspond to correlation: Positive correlation (Red); Negative correlation (Blue); No correlation (White). (**C**) Function enrichment analysis of each developmental stages specific modules. Length of bars indicate the significance (−log10 transferred *P*-value).

Because there never was a study describe the expression profiling of lincRNAs during porcine PED, and functional research about these lincRNAs were also limited. Here, we firstly filter the low variance lincRNA and mRNAs across each embyronic developmental stages, and then performed weighted gene co-expression network analysis (WGCNA) to investigate the potential roles of lincRNAs in porcine PED^19^,^36^. We identified 23 co-expression modules through unsupervised and unbiased clustering (Fig. 4A and see Supplementary Fig. S2). Notably, 5 out of 23 co-expression modules showed developmental stages specific (correlation >0.7, *P*-value < 10^−4^) (Fig. 4B), which probably represent core gene net-works operating in each transitional stage. In total, 3723 genes (3105 mRNAs and 618 lincRNAs) were part of porcine 1-cell to morula stage-specific modules (see Supplementary Fig. S3). To further predict the function of lincRNAs in porcine PED, we performed GO enrichment analysis with the mRNAs in each stage-specific modules. As expect, we found that the modules in the 4- to 8-cell transition corresponding to zygotic genome activation (ZGA) in pig were enriched for transcription regulation, epigenetic regulation and cell cycle (Fig. 4C and see Supplementary Table S3). These results suggest that the lincRNAs in these two modules might play important roles during the ZGA process through various regulation mechanism, such as the *cis*-acting that was mentioned above.

### Identification of hub lincRNAs in porcine pre-implantation embryonic development

**Figure 5:**
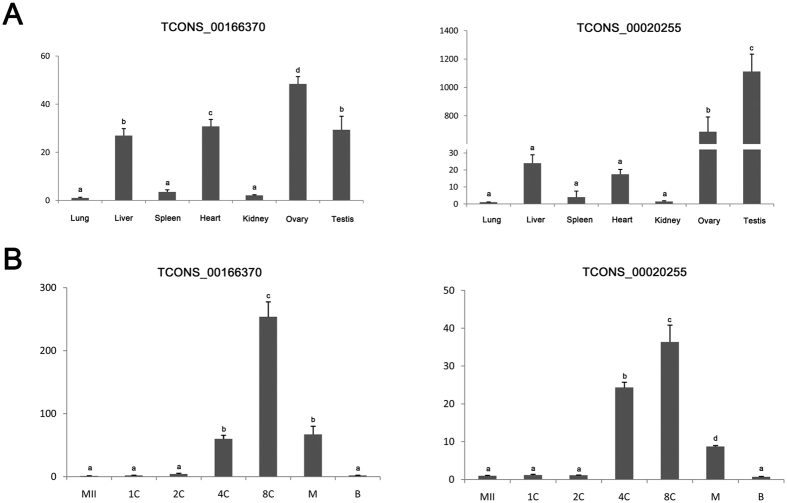
The expression of two hub lincRNAs from 4-cell stage-specific module in different tissues and PED. (**A)** qPCR results showed that the two selected lincRNAs were mainly expressed in reproductive tissues, such as the testis and ovary. (**B**) The expression level of *TCONS_00168370* and *TCONS_00020255* increase at 4-cell stage and reached a peak at the 8-cell stage. ACTB served as control. Results are presented as mean values ± SEM. Different letters indicate significant differences (*p* < 0.05).

Hub genes are centrally located in their respective modules and may thus reflect the core functions of the network^37^. To identify hub lincRNAs during porcine PED, we used WGCNA measure of intramodular gene connectivity, and extracted the top 100 as hub genes in each stage-specific modules (see Supplementary Table S4). We next examined the expression of ten selected lincRNAs obtained from the hub genes sets in 4-cell stage-specific module in seven tissues of pig through quantitative realtime polymerase chainreaction (qRT-PCR) analysis, and found that these lincRNAs as a whole were expressed in reproductive tissues (Fig. 5A and see Supplementary Fig. S4). Then, we found two lincRNAs: *TCONS_00166370* and *TCONS_00020255* with a high expression in ovary displayed a sharp activation tend from 4-cell stage, and declined rapidly after 8-cell stage, which correspond to our prediction (Fig. 5B). In addition, we further validated the transcriptional direction of the two lincRNAs through Strand Specific RT-PCR (SSRT) analysis (see Supplementary Fig. S5). The reliability of SSRT-PCR results were confirmed by sequencing, suggesting the robustness of our results. Though little is known about how these lincRNAs involved in the porcine PED, the identified hub lincRNAs catalog would serve as a valuable resource for further functional researches.

## Discussion

Despite recent genome-wide studies have revealed tens of thousands of lincRNAs^1^,^2^,^3^,^4^, porcine lincRNAs were poorly annotated. Nevertheless, with the advancement of high-throughput technologies for large-scale expression, RNA-seq has accelerated the discovery and characterization of lincRNAs to an unprecedented degree. In this article, we perform comprehensive analysis of porcine lincRNAs based on published RNA-seq data sets, and identified 7,618 novel lincRNAs from 4,776 gene loci. More importantly, as for the first study of lincRNAs in porcine PED, we constructed a weighted gene co-expression network and investigated the hub lincRNAs that may be likely to be key in driving porcine PED.

During the past 5 years, several tools heavily relied on sequence alignment, such as CPC^14^ and PORTRAIT^38^, have been developed to predict the coding potential based on known protein database. However, most lincRNA discovered tend to be lineage specific and less conserved^27^,^39^. For example, only 993 of 8195 human lncRNAs have orthologous transcripts in other species^27^. Therefore, it is hard to define the coding potential using these annotation-based approaches. Considering the incomplete annotation in pig, herein we performed genome-wide characterization of novel lincRNAs using two signature tools independent of known annotations. As results, our newly identified lincRNAs in pig shared many characteristics with those in other mammalian species^3^,^16^,^17^,^27^,^28^,^29^. They are lower in exon number, longer in exon, shorter in whole transcripts, lower in expression level and more specific in expression patterns than protein coding transcripts. In particular, several studies indicated that some lincRNAs can regulate gene expression of their neighborhood in *cis*^32^,^33^. According to analysis of correlations of expression between lincRNA and their coding neighboring protein-coding genes revealed that, we found lincRNAs have highly positive correlation with neighboring genes.

Research on preimplantation development, especially the cleavage stage development, is important for both reproductive biology and regenerative medicine. Besides, understanding the nature of reprograming and totipotency of PED will enlighten the research on and utilization of ESCs and iPSCs. However, PED is a extremely complex process, where a series of important distinctive developmental events happened, such as maternal-zygotic transition^40^, ZGA^41^, and segregation of inner cell mass and trophectoderm^42^,^43^,^44^. Therefore investigating of the molecular network of lincRNAs during PED are vitally important. Although many lincRNAs have been indicated to play important roles in a variety of biological processes. However, to date, there was only one sutdy reports an ZGA-specific lncRNA named *pancIl17d* palying essential roles in mouse PED, and shows that *pancIl17d* enhance the transcription of its neighboring protein-coding gene *Il17d* in *cis* for driving subsequent PED. This is the first time to analyzed the lincRNAs in porcine PED, and these developmental stage-specific modules identified by WGCNA suggested the diverse functions of lincRNAs in porcine PED. Procine ZGA generally occurs at the 4-cell stage, and go functional annotation analysis of the 4-cell specific module showed that they were mainly involved in the “RNA processing” and “cell cycle”, which correspond to the previous finding that a set of lincRNAs transcribed within cell-cycle promoter of human^10^.

In the hub genes networks, we found two lincRNAs were mainly expressed in reproductive tissues and displayed a sharp activation from 4-cell stage. Previous studies have demostrated that many lincRNAs could exert their function through related mRNA which can play pivotal roles in various biological processes^7^,^45^,^46^. Remarkably, we identified several overlap hub genes which have high correlation to *TCONS_00166370* and *TCONS_00020255* (Supplementary Table S6). For example, *Lin28* (weight = 0.76 to *TCONS_00166370* and 0.75 to *TCONS_00020255*) is consistently identified as a key gene in multiple human and mouse ZGA networks, and its deficience leading to a developmental arrest at the 2-cell stage to 4-cell stage in mouse^47^. Furthermore, *Cdc7* (weight = 0.76 to *TCONS_00166370* and *TCONS_00020255*), an S-phase-promoting kinase, is also required for mouse PED^48^. Together, these results demonstrate that the two lincRNAs might play crucial roles in procine PED.

In summary, we performed comprehensive analysis of porcine lincRNAs, and provide the first lincRNA profiles of procine PED. These identified lincRNAs in pig show many similar characteristics with those in other mammalian species. WGCNA analysis suggested many lincRNAs in porcine PED are involved in cell cycle regulation, transcription and epigenetic to regulate the process of PED. As the role of lincRNAs in pigs have not yet been fully identified and understood, this work provides a valuable resource for further analyses. Moreover, the putative lincRNAs in stage-specific modules could have important roles in porcine PED and deserve further functional studies.

## Materials and Methods

### Ethics Statement

All studies involving animals were conducted according to regulation approved by the Standing Committee of Heilongjiang People’s Congress, P. R. China. Sample collection was approved by the ethics committee of Northeast Agricultural University. Animals were humanely sacrificed as necessary to ameliorate suffering.

### Datasets used in this study

The data included five RNA-seq data sets was down-loaded from the NCBI SRA database^13^,^20^,^21^,^23^,^42^. The accession numbers and detailed information of the RNA-seq data are listed in Supplementary Table S1.

### RNA-seq data analysis

Reads were aligned to sus scrofa 10.2 genome using TopHat version 2.0.9 described in ref. 27. Mapped reads from TopHat for each sample were assembled used Cufflinks vision 2.1.1. The multiple assembled transcript files (GTF format) for different sample were then merged together to produce a unique transcriptome set using the Cuffmerge utility provided by Cufflinks package^24^.

### LincRNA detection pipeline

To identify lincRNAs in pig, we designed an analysis pipeline to minimize false positives and maximize the number of lincRNA transcripts, including the following five steps: (1) we used Cuffcompare to compare our merged transcriptome with annotation in Ensembl databases, and removed potential known transcripts; (2) filter transcripts that are shorter than 200 nt; (3) select transcripts that are more than 2 exon; (4) Keep only transcripts that are located at least 500 bp away from any protein-coding genes or house-keeping ncRNAs genes annotated in the Ensembl Sus scrofa10.2 gene set (GTF); (5) filter putative lincRNA transcripts by coding potential using the CNCI and CPAT software^25^,^26^, which are independent of known annotations and have been proved the best effective lncRNA identification.

### Tissue-specific assessment

We used a probability distribution distance metric related to Jensen-Shannon divergence (JSD) to quantify tissue specificity^27^. The metric quantifies the similarity between expression pattern in a given sample and an extreme pattern that represents that a transcript is expressed in only one tissue. The specificity score is defined as 1-(JSdist(p,q)), where p is the density of expression (probability vector of log10(FPKM +1)) of a given gene across all conditions,and q is the unit vector for that condition (ie. perfect ex-pression in that particular condition), while JSdist is a function that used to calculate pairwise Jensen-Shannon distances between columns. We use max JS score of a transcript to represent the expression specificity of it ref. 16.

### Correlations of neighbouring gene

We focused our attention on pairwise correlations of expression involving neighboring genes, which the minimal distance <10 kb and ignore the direction of two genes. Pearson correlation of two neighbours was calculated with log2-normalization (after addition of 0.05) of raw expression level (FPKM).

### Weighted gene co-expression network analysis

Before the WGCNA analysis, we performed the following pretreatment of the expression matrix: (1) we removed the genes that max expression level (FPKM) <0.05 acorss the porcine PED samples; (2) select transcripts that the variance are the top 75%; (3) final expression matrix was constructed with log2-normalization (after addition of 1) of raw expression level (FPKM).

R package “WGCNA” was used to construct the weighted gene co-expression network. First, a signed weighted correlation network was constructed by creating a matrix of pairwise correlations between all pairs of genes across the porcine PED samples^36^. Second, the adjacency matrix was constructed by raising the co-expression measure, 0.5 + 0.5 × correlation matrix, to the power = 13. The power of 13 is the soft-threshold of correlation matrix and makes the adjacency network exhibit approximate scale-free topology (R-squared = 0.9). Based on the resulting adjacency matrix, we transformed the adjacency matrix to topological overlap matrix (TOM)^49^. Genes with highly similar co-expression relationships were clustered together. To defined modules as branches, we performed the Dynamic Tree Cut algorithm^50^ with default parameters to cut the hierarchal clustering tree. We summarized the expression profile of each module by its first principal component (module eigengene). Modules whose module eigengenes (correlation >0.7) were merged together.

### Identification of hub genes

The module membership (also known as module eigengene based connectivity, kME) of each genes was calculated based on the module eigengenes. Specifically, the module membership for gene i with respect to module *q* is defined as follows *MM*^*q*^(*i*) = *cor(x(i), E*^*q*^), where *x(i*) is the expression profile of gene *i* and *E*^*q*^ is the eigengene of module *q*. Therefore the score of *MM* represents the extent of a gene close to a given module. The advantage of using a correlation to quantify module membership is that this measure is naturally scaled to lie in the interval [−1, 1] and a corresponding statistical significance measure (*P* value) can be easily computed. Genes with highest module membership values are referred to as hub genes. Hub genes are centrally located in their respective modules and may thus reflect the core functions of the network^37^.

### Function enrichment analysis

The Database for Annotation, Visualization and Integrated Discovery (DAVID) was a frequently-used bioinformatics resources for GO functional annotation. First, we upload gene lists to DAVID. And then, after selecting identifier for thes genes (In this work, we select “ENSEMBL_GENE_ID”). Biological process, molecular fuction and cellular component terms was seleted as background gene sets respectively. Fisher Exact test was used to measure gene-enrichment in background annotation terms^51^.

### Porcine embryo collection and culture

All experiments were were performed according to the guid-lines of The State Key Laboratory Animal Care and Use Committee. The procedure for porcine IVF has been described previously^52^. Briefly, freshly ejaculated sperm-rich fractions were collected from fertile boars. Following short incubation at 39 °C, semen was resuspended and washed three times in DPBS supplemented with 0.1% (w/v) BSA via centrifugation at 1500 g for 4 min. Spermatozoa concentrations were measured using a hemocytometer, and the proportion of motile sperm determined. Next, spermatozoa were diluted with modified Tris-buffered medium (mTBM) to an optimal concentration. Cumulus-free oocytes were washed three times in mTBM. Approximately 30 oocytes were inseminated in 50 ml mTBM at a final sperm concentration of 3 × 10^5^/ml for 5 h. Embryos were cultured in porcine zygote medium-3 (PZM-3) at 39 °C in 5% CO_2_ in air. Embryos were collected after IVF at the following time points: 1-cell stage (24 hours), 2-cell stage (40–45 hours), 4-cell stage (65–72 hours), 8-cell stage (84–90 hours), morlua stage (108–115 hours) and blastocyst stage (156–160 hours). Besides, the oocytes were collected at 42 h *in vitro* maturation. For qPCR, about 50 embryos of each stage were used.

### Real-time RT-PCR analysis

Total RNA was extracted using the PureLink^TM^ Micro-to-Midi System (Invitrogen) according to the manufacturer’s instructions, and reverse transcription was used to generate cDNAs using the PrimeScript^TM^ RT Reagent kit (TaKaRa). Real time PCR was performed using SYBR Premix Ex Taq^TM^ (TaKaRa) and the 7500 Real-Time PCR System (Applied Biosystems). The reaction parameters were 95 °C for 30 s followed by 40 two-step cycles of 95 °C for 5 s and 60 °C for 34 s. All the primer pairs used to PCR amplification were shown in Supplementary Table S5. Ct values were calculated using Sequence Detection System software (Applied Biosystems), and the amount of target sequence normalized to the reference sequence was calculated as 2^−ΔΔCt^.

### Statistical analysis

Statistical analysis was performed using SPSS 19.0 for MicroSoft^TM^ Windows. Data are presented as means ± SEM. The Least Significant Difference method was employed for multiple comparisons. Data were considered statistically significant at *p* < 0.05.

## Additional Information

**How to cite this article**: Li, J. *et al*. Identification and functional analysis of long intergenic noncoding RNA genes in porcine pre-implantation embryonic development. *Sci. Rep.* **6**, 38333; doi: 10.1038/srep38333 (2016).

**Publisher's note:** Springer Nature remains neutral with regard to jurisdictional claims in published maps and institutional affiliations.

## Supplementary Material

Supplementary Information

Supplementary Dataset 1

Supplementary Dataset 2

Supplementary Dataset 3

Supplementary Dataset 4

Supplementary Dataset 5

Supplementary Dataset 6
