# Supplementary material for: Identification and functional analysis of long intergenic noncoding RNA genes in porcine pre-implantation embryonic development
Source: Sci Rep. 2016 Dec 6;6:38333. doi: 10.1038/srep38333 (PMC5138625; doi:10.1038/srep38333)
Supplement: Supplementary Information [file srep38333-s1.pdf]

Identification and functional analysis of long intergenic noncoding RNA genes in porcine pre-implantation embryonic development

Jingyu Li[1], Zhengling Gao[1], Xingyu Wang[2], Hongbo Liu[2], Yan Zhang[2*], Zhonghua Liu[1*]

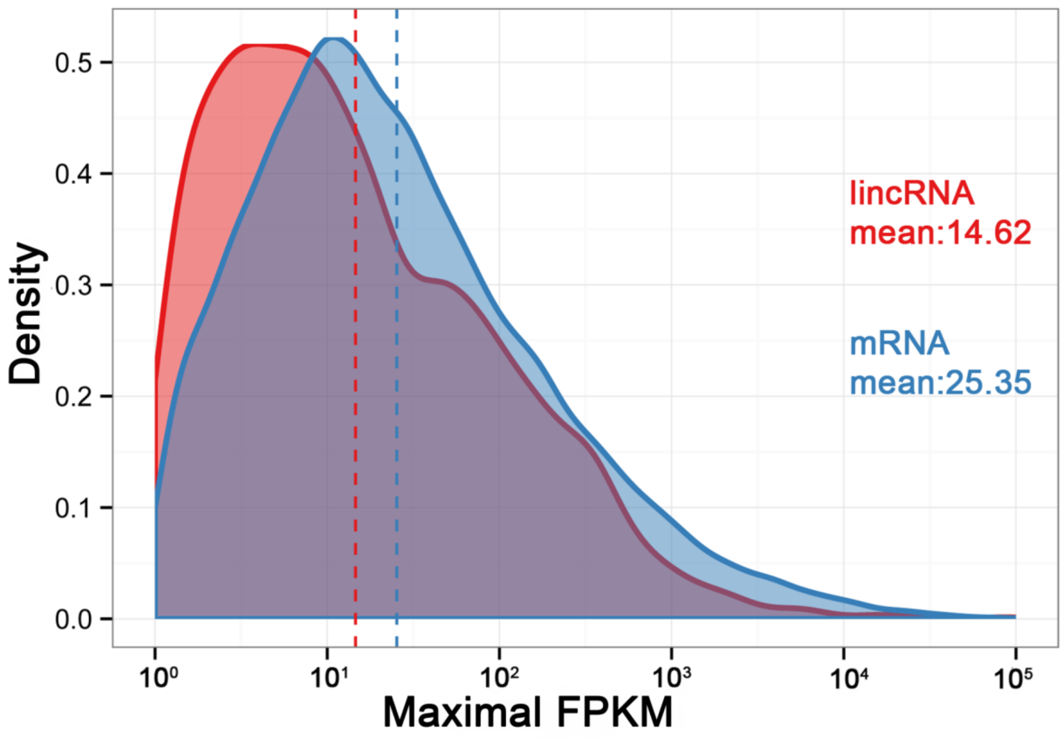

Supplementary Figure S1. Distribution of maximal expression level of lincRNAs and mRNAs across five RNA-seq datasets.

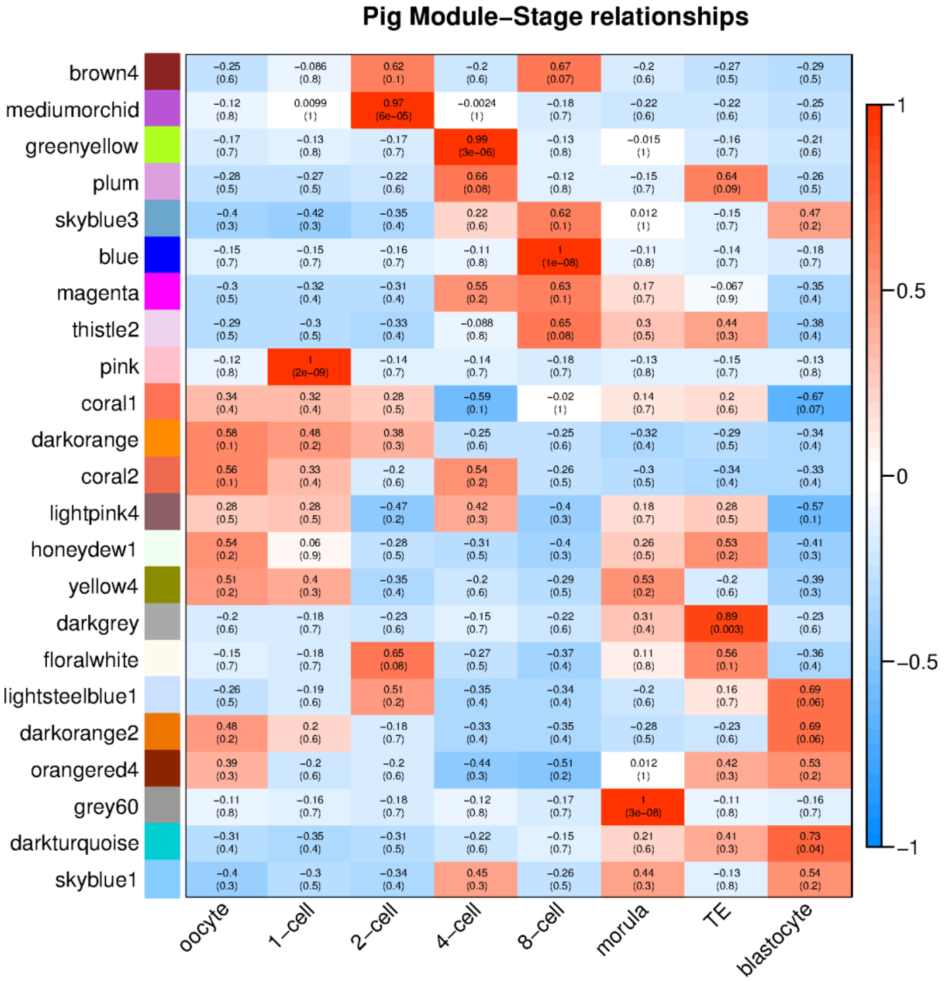

Supplementary Figure S2. Module development stage correlation. Each row corresponds to a module eigengene, column to each development stage. Each cell contains the corresponding correlation and *p*-value. The table is color-coded by correlation according to the color legend.

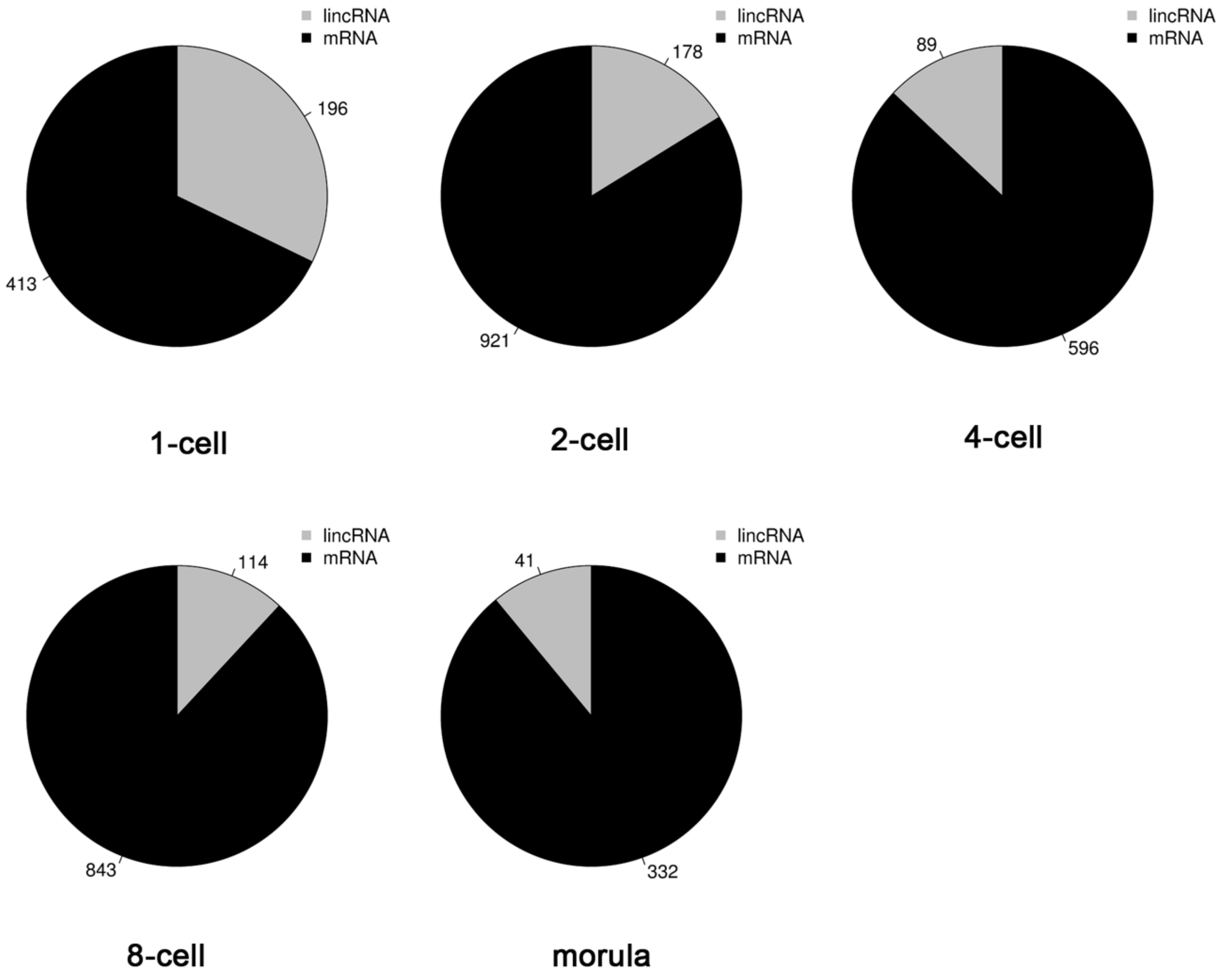

Supplementary Figure S3. The ratio of mRNAs and lincRNAs in module.

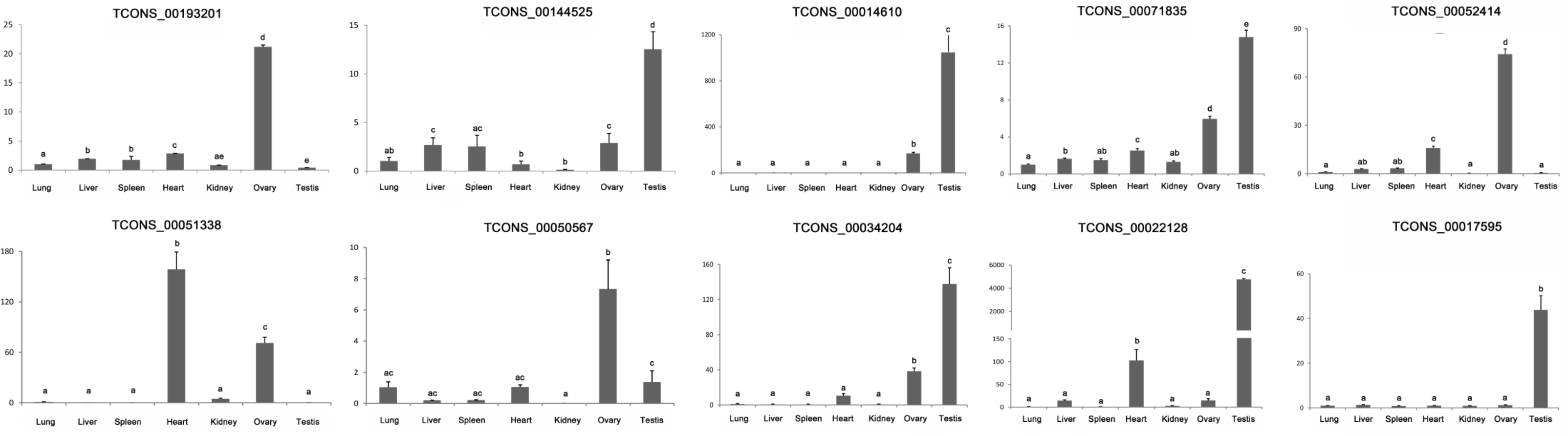

Supplementary Figure S4. The expression of eight hub lincRNAs from 4-cell stage-specific module in porcine different tissues. Results are presented as mean values ± SEM. Different letters indicate significant differences (*p*<0.05).

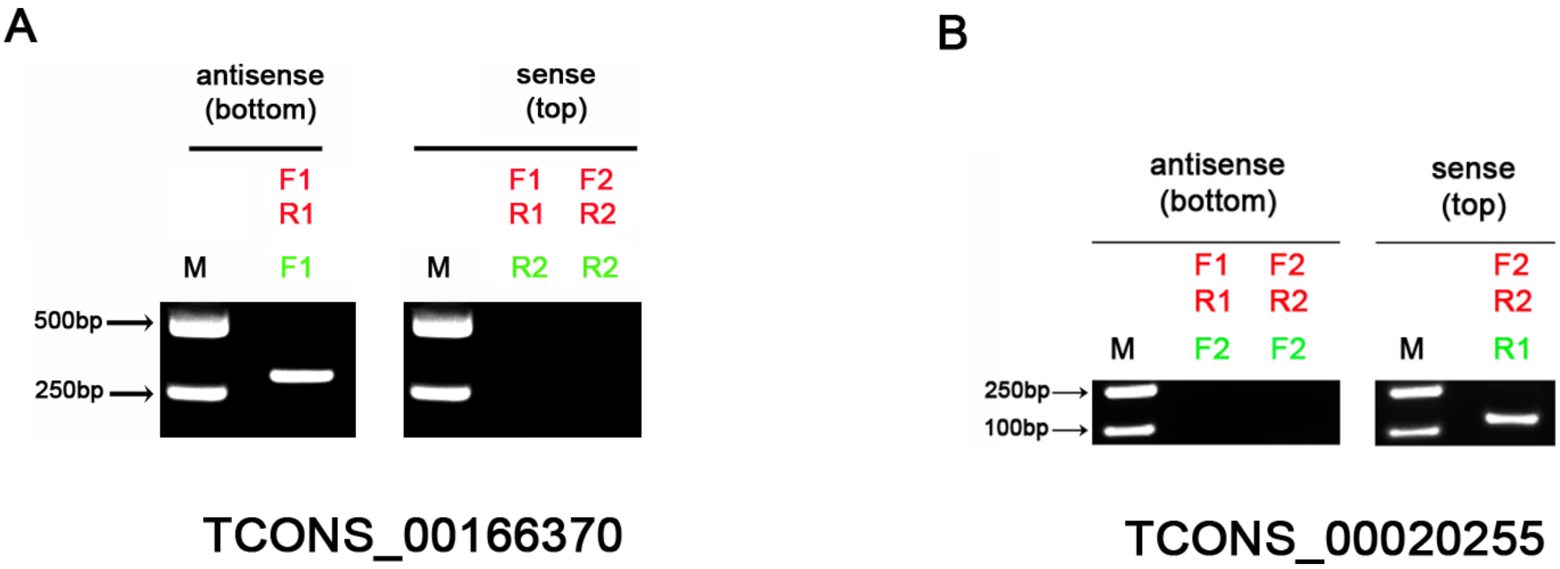

Supplementary Figure S5. SSRT-PCR result of *TCONS_00166370* and *TCONS_00020255*. It shows that *TCONS_00166370* is transcribed from the bottom strand, and *TCONS_00020255* are the from the top strand.
