# Supplementary material for: Identification and functional analysis of long intergenic noncoding RNA genes in porcine pre-implantation embryonic development
Source: Sci Rep. 2016 Dec 6;6:38333. doi: 10.1038/srep38333 (PMC5138625; doi:10.1038/srep38333)
Supplement: Supplementary Dataset 1 [file srep38333-s2.doc]

**Identification and functional analysis of long intergenic noncoding RNA genes in porcine pre-implantation embryonic development**

Jingyu Li^1^, Zhengling Gao^1^, Xingyu Wang^2^, Hongbo Liu^2^, Yan Zhang^2*^, Zhonghua Liu^1*^

**Supplementary Table S1 Details of RNA-seq data**

| SRA ID | Platform | Run | | Tissue/Cell line**s** | Mapped reads | Reference |
| --- | --- | --- | --- | --- | --- | --- |
| SRA143032 | SOLiD | SRR1181484 | | oocyte | 10975851 | [^1^](#_ENREF_1) |
|  |  | SRR1181599 | | 1-cell | 31446457 |  |
|  |  | SRR1185956 | | 2-cell | 20269213 |  |
|  |  | SRR1185984 | | 4-cell | 25312481 |  |
|  |  | SRR1185988 | | 8-cell | 28196828 |  |
|  |  | SRR1186927 | | morula | 25649186 |  |
|  |  | SRR1186928 | | blastocyte | 14351687 |  |
|  |  | SRR1186929 | | TE | 17127030 |  |
| SRA065859 | Illumina | SRR653843 | | heart | 81271901 | [2](#_ENREF_2) |
|  |  | SRR653844 | | liver | 14001364 |  |
|  |  | SRR653845 | | lung | 22285185 |  |
|  |  | SRR653846 | | kidney | 22170696 |  |
| SRA031272 | Illumina | SRR167669 | | liver | 31358981 |  |
|  |  | SRR167670 | | abdominal fat | 30199929 | [3](#_ENREF_3) |
|  |  | SRR167671 | | longissimus dorsi muscle | 28257759 |  |
|  |  | SRR167672 | | liver | 32020025 |  |
|  |  | SRR167673 | | abdominal fat | 30444965 |  |
|  |  | SRR167674 | | longissimus dorsi muscle | 28671763 |  |
| SRA093372 | Illumina | SRR934623 | | endometrial | 27102896 | [4](#_ENREF_4) |
|  |  | SRR934624 | | endometrial | 28165630 |  |
|  |  | SRR934625 | | endometrial | 26582193 |  |
|  |  | SRR934626 | | endometrial | 26135704 |  |
|  |  | SRR934627 | | endometrial | 29044601 |  |
|  |  | SRR934628 | | endometrial | 30298458 |  |
|  |  | SRR934629 | | endometrial | 28333255 |  |
|  |  | SRR934630 | | endometrial | 29224121 |  |
| SRA092777 | Illumina | SRR931785 | sperm | | 30483450 | [5](#_ENREF_5) |
|  |  | SRR931786 | sperm | | 26198523 |  |
|  |  | SRR931787 | sperm | | 29414764 |  |
|  |  | SRR931788 | sperm | | 21475053 |  |

1 Zhou, Z. Y. *et al.* Genome-wide identification of long intergenic noncoding RNA genes and their potential association with domestication in pigs. *Genome Biol Evol* **6**, 1387-1392, doi:10.1093/gbe/evu113 evu113 [pii] (2014).

2 Li, M. *et al.* Genomic analyses identify distinct patterns of selection in domesticated pigs and Tibetan wild boars. *Nat Genet* **45**, 1431-1438, doi:10.1038/ng.2811 ng.2811 [pii] (2013).

3 Chen, C. *et al.* A global view of porcine transcriptome in three tissues from a full-sib pair with extreme phenotypes in growth and fat deposition by paired-end RNA sequencing. *BMC Genomics* **12**, 448, doi:10.1186/1471-2164-12-448 1471-2164-12-448 [pii] (2011).

4 Samborski, A. *et al.* Transcriptome changes in the porcine endometrium during the preattachment phase. *Biology of reproduction* **89**, 134, doi:10.1095/biolreprod.113.112177 biolreprod.113.112177 [pii] (2013).

5 Bruggmann, R., Jagannathan, V. & Braunschweig, M. In search of epigenetic marks in testes and sperm cells of differentially fed boars. *PloS one* **8**, e78691, doi:10.1371/journal.pone.0078691 PONE-D-13-22313 [pii] (2013).
